# Reprogramming diminishes retention of *Mycobacterium leprae* in Schwann cells and elevates bacterial transfer property to fibroblasts

**DOI:** 10.12688/f1000research.2-198.v3

**Published:** 2013-11-14

**Authors:** Toshihiro Masaki, Aidan McGlinchey, Simon R. Tomlinson, Jinrong Qu, Anura Rambukkana

**Affiliations:** 1 MRC Center for Regenerative Medicine, University of Edinburgh, Edinburgh, EH16 4UU, UK; 2 Center for Neuroregeneration, University of Edinburgh, Edinburgh, EH16 4UU, UK; 3 Center for Infectious Diseases, University of Edinburgh, Edinburgh, EH16 4UU, UK; 4 Laboratory of Bacterial Pathogenesis and Immunology, Rockefeller University, New York, 10065, USA

## Abstract

**Background:** Bacterial pathogens can manipulate or subvert host tissue cells to their advantage at different stages during infection, from initial colonization in primary host niches to dissemination. Recently, we have shown that *Mycobacterium leprae* (ML), the causative agent of human leprosy, reprogrammed its preferred host niche de-differentiated adult Schwann cells to progenitor/stem cell-like cells (pSLC) which appear to facilitate bacterial spread. Here, we studied how this cell fate change influences bacterial retention and transfer properties of Schwann cells before and after reprogramming.

**Results:** Using primary fibroblasts as bacterial recipient cells, we showed that non-reprogrammed Schwann cells, which preserve all Schwann cell lineage and differentiation markers, possess high bacterial retention capacity when co-cultured with skin fibroblasts; Schwann cells failed to transfer bacteria to fibroblasts at higher numbers even after co-culture for 5 days. In contrast, pSLCs, which are derived from the same Schwann cells but have lost Schwann cell lineage markers due to reprogramming, efficiently transferred bacteria to fibroblasts within 24 hours.

**Conclusions:** ML-induced reprogramming converts lineage-committed Schwann cells with high bacterial retention capacity to a cell type with pSLC stage with effective bacterial transfer properties. We propose that such changes in cellular properties may be associated with the initial intracellular colonization, which requires long-term bacterial retention within Schwann cells, in order to spread the infection to other tissues, which entails efficient bacterial transfer capacity to cells like fibroblasts which are abundant in many tissues, thereby potentially maximizing bacterial dissemination. These data also suggest how pathogens could take advantage of multiple facets of host cell reprogramming according to their needs during infection.

## Introduction

The peripheral nervous system (PNS) is the preferred residence for one of the oldest bacterial pathogens known to mankind, *Mycobacterium leprae *(ML). It causes human leprosy, which is a chronic neurological disease and still remains a public health problem ^1^. The large gaps in our understanding of the infectious process have halted the progress towards developing effective early diagnostics and therapeutics for the management of nerve damage, the pathological hallmark of the disease. The distinctive peripheral nerve involvement is directly associated with the remarkable capacity of ML to invade the supporting glial cells of the adult PNS, Schwann cells ^2^. Because peripheral nerves are a privileged site and are therefore protected from immune cells due to blood nerve barrier, Schwann cells provide a safer niche for ML survival, propagation and initial colonization.

One of the primary functions of Schwann cells is to synthesize the myelin sheath around axons that deliver rapid nerve conduction ^3^. Despite the terminal differentiation of Schwann cells to a highly sophisticated myelinated phenotype, these mature Schwann cells show unprecedented plasticity; they can switch off their myelin program in response to injury and acquire a de-differentiated state resembling an immature phenotype, but maintain Schwann cell lineage properties ^4, 5^. ML appear to take advantage of this natural property and induce myelin damage (demyelination) as an adaptive mechanism to generate similar de-differentiated Schwann cells that are more favourable for bacterial colonization and manipulation ^6^. We have recently shown that once infected these de-differentiated Schwann cells purified from adult nerves, they undergo a reprogramming process and convert to highly immature progenitor/stem cell-like cells (pSLC) that are more suitable for spreading infection to other tissues either by direct re-differentiation or through macrophages ^7^. However, it is not clear if fibroblasts, which are ubiquitous in many tissues, could play an intermediary role by receiving ML and if so how pSLC differ from non-reprogrammed Schwann cells in terms of bacterial transfer.

In this study, using primary mouse fibroblasts as a model, we assessed the bacterial transfer capacity of Schwann cells before and after reprogramming. We showed that before reprogramming, lineage committed Schwann cells possess a high bacterial retention capacity. However, after reprogramming, which downregulated all Schwann cell lineage markers and myelin markers, Schwann cells lose bacterial retention and acquire an efficient bacterial transfer property to co-cultured fibroblasts. These findings show an example of how a bacterial pathogen could use an induced cell fate change to suit its own ends during different stages of infectious process.

## Materials and methods

### Preparation of primary Schwann cells from adult peripheral nerves

Adult CD-1 mice (4–6 week old, ICR strain code: 022) and 6–8 week old GFP mice that constitutively express eGFP (strain: C57BL/6-Tg [ACTB-EGFP]1Osb/J, stock: 003291) were purchased from Charles River and Jackson Laboratories (Bar Harbor, ME). Animals were maintained at the Rockefeller University animal facilities in pathogen free cages. Institutional Animal Care and Use Committee (IACUC) at the Rockefeller University approved all animal procedures and ethical issues. For isolating Schwann cells, 6–8 mice were used and cells were then purified using magnetic cell sorting system and FACS sorting using anti-p75 antibody (AB1554, Millipore, USA) as described ^7^.

### ML infection and reprogramming of mouse Schwann cells

Purified Schwann cells were grown in collagen coated T25 or T75 flasks (BD Biosciences, NJ, USA) and infected with ML and reprogrammed cells were generated according to our previous protocol ^7^. *In vivo*-grown viable ML derived from nude-mouse footpads were prepared as described previously ^8^. Briefly, p75+/Sox10+/Sox2+ Schwann cells purified from adult GFP-mice and wild type were infected with ML and maintained in Schwann cell media as described in details ^7^. At day 3 post-infection, Schwann cells maintain p75+/Sox10+/Sox2+ and all other Schwann cell phenotype, and these infected cells were used in this study. In parallel, infected cells were incubated for four weeks and then FACS sorted for p75-cells; their phenotypes were confirmed as p75-/Sox10-/Sox2+, and reprogrammed cells were isolated from this population based on their ability to grow in mesenchymal stem cell media (StemCell Inc, Vancouver, Canada) and were termed as progenitor/stem-like cells (pSLC) ^7^. pSLC were transduced with CopGFP-CDH-MSCV-cG reporter vector ^9^ obtained from System Biosciences (CA) and stable expression of copGFP in pSLC was performed as reported earlier ^7^. GFP+pSLC were re-infected with ML in order to maintain similar bacterial load as with Schwann cells at day 3 post-infection, since reprogramming process that accompanies long incubation and cell proliferation causes bacterial dilution.

### Preparation of primary neural and skin fibroblasts

Neural fibroblasts were prepared from adult CD-1 male mice (4–6 week old, ICR strain code: 022) at the same time when Schwann cells were prepared from these mice ^1^. In brief, peripheral nerve tissues were isolated in MEM (Invitrogen) and then digested with 0.125% trypsin (Invitrogen)/0.05% EDTA and 0.1 mg/ml collagenase I (Worthington Biochemical) and passed through 100 micron mesh nylon filter (BD Falcon). Cells were collected and seeded on T25 flask cultured in 10% FCS medium. Propagated cells negative for p75NGFR Schwann cell surface marker were separated by magnetic cell sorting system followed by FACS sorting using anti-p75 antibody (AB1554, Millipore, USA). The purified p75-negative cells, which were also negative for Sox2 and Sox10 were used for co-culture experiments. Skin fibroblasts were prepared from adult wild-type mouse skin using a similar protocol described in detail previously ^10^.

### Bacterial retention and transfer determination and microscopy

**Figure 1.**
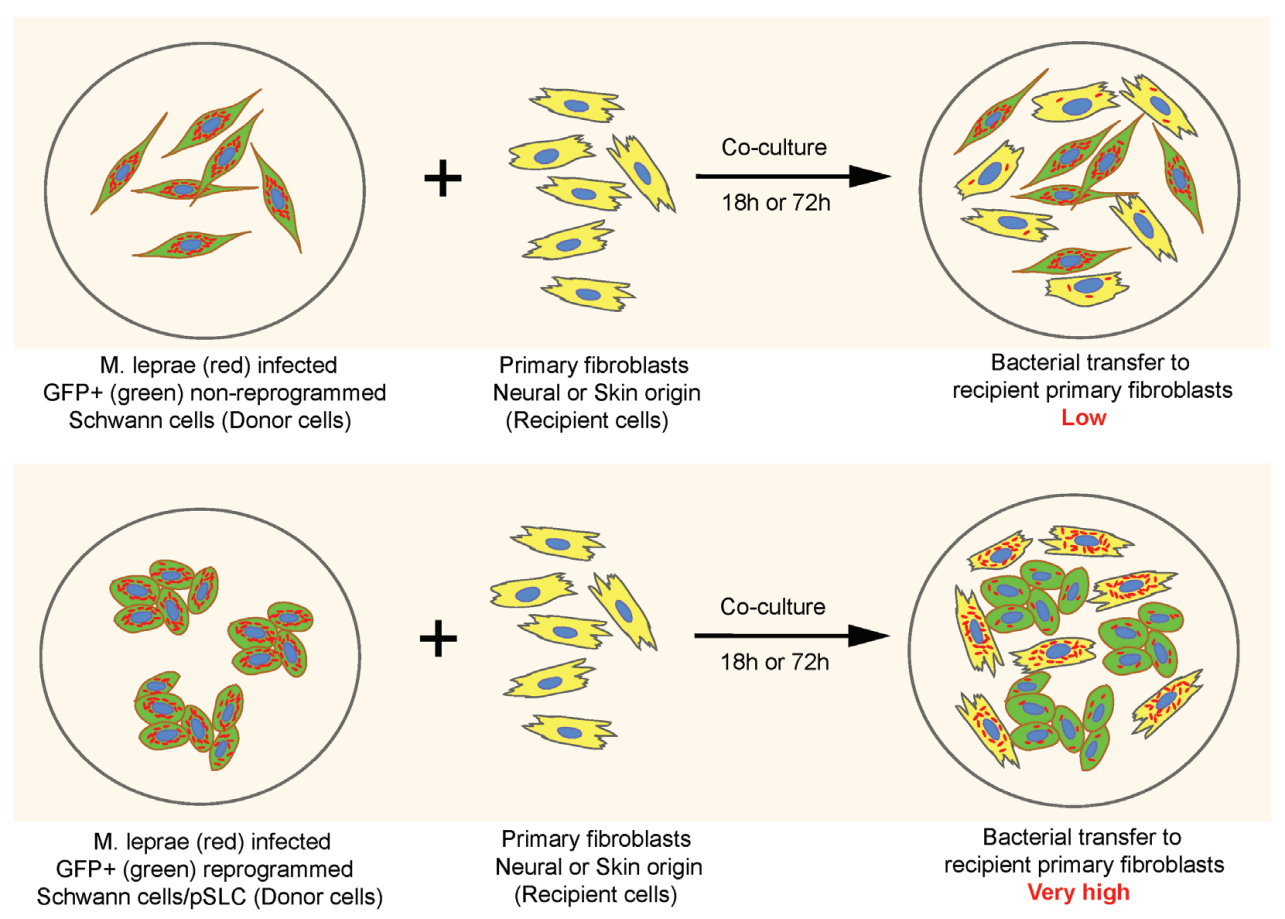
Schematic illustrating the summary of the experimental approach and the results. GFP+ non-reprogrammed and reprogrammed Schwann cells (pSLC) that are infected with ML (red bacteria in green cells) are co-cultured with exogenously added primary fibroblasts (yellow) derived from adult peripheral nerves or skin. Evaluation of bacterial transfer to recipient fibroblasts from GFP+ cells after 18h and 72h revealed an effective bacterial retention in non-reprogrammed cells (top) and a significant ML transfer to fibroblasts from reprogrammed Schwann cells (bottom).

Figure 1 summarizes the experimental scheme described below. Day 3-infected GFP+Schwann cells and re-infected GFP+pSLC that carry >50–100 ML per cell (infection efficiency is >90%) were first washed to remove any extracellular ML and then co-cultured with GFP-negative fibroblasts for 18 hours, 3 and 7 days. Pre-evaluated culture medium containing DMEM with 6% serum (HyClone, USA) that suits short-term co-cultures, Schwann cell-fibroblasts and pSLC-fibroblasts co-culture combinations were selected. Although we used this medium for short-term cultures for quantification, both mesenchymal media without supplements and Schwann cell media were also supported cell growth for a short period with no apparent phenotypic change in Schwann cells and pSLC.

Also, a possible release of bacteria to the culture media from infected pSLC was determined by using pooled culture media from day 2-cultured pSLC. Cell supernatants from infected pSLC were centrifuged at 15,000 rpm for 20 minutes, and pelleted ML were re-suspended in 50 µl PBS, placed on glass coverslips and fixed and immunolabelled with anti-PGL-1 antibody as described below. As a positive control for ML, a suspension of ML in DMEM (1x10 ^6^ ML/ml) was used. Whereas ML suspension showed numerous PGL-1+ ML, hardly any bacteria were detected from supernatants from pSLC.

Bacterial transfer to non-GFP fibroblasts from GFP+ cells or bacterial retention within GFP+ cells were evaluated by immunolabeling of fixed cultures using ant-PGL-1 antibody ^7^ against GFP+/- detection. PGL-1/ML+ cells, both GFP+ and non-GFP cells, containing >50 ML per cells were quantified 18 hours, 3 and 5 days after addition of fibroblasts. Data were analyzed for statistical significance using Student’s t test or by regression analysis (SigmaPlot). Paired sample analysis with P values <0.01 were considered as significant.

Immunofluorescence was performed as described ^7^. In brief, cells were fixed with 4% formaldehyde (Sigma) for 10 min at room temperature and 100% methanol (Sigma) for 10 minutes at -20ºC. The samples were washed with PBS, blocked with 10% goat serum and then incubated with primary antibody followed by secondary antibody conjugated with Alexa Fluor 488 or 594 (Invitrogen) as described; details of anti-p75 and anti-PGL-1 and secondary antibodies have been described previously ^7, 14^. Assays for apoptotic cells in co-cultures were performed using TUNEL assay kit (R&D Systems) according to manufactures instructions as described previously ^7, 15^. Images were captured with Nikon Eclipse 2100 microscopy.

### Gene-Expression analyses

Gene-Expression Analyses were performed using Affymetrix mouse gene chips according to Affymetrix protocol as described previously ^7^. In brief, total RNA was isolated from uninfected/control Schwann cells, 3 days post-infection and pSLC derived from day 28-infected Schwann cells using RNeasy columns (QIAGEN). Affymetrix Test3 arrays and mouse genome MG-430A2 arrays were probed with the cRNA prepared by reverse-transcription of the total RNA. Microarray data were processed by the Robust Multichip Average (RMA) method ^11^. The R statistical programming language was used (version 2.15.2), in tandem with the Bioconductor Analysis suite (version 2.12) ^12^. The resulting probe mRNA detection data was searched and selected for markers relating to Schwann cell lineage/myelination, based on published literature 3– 7. This list of probes contained duplicate probes for the same gene symbol and so was trimmed to include only one representative probe per gene symbol, both for clarity and due to space constraints. The data were presented in the form of heatmap representing expression of the genes (absolute log2 expression values) associated with these probes in each replicate at each time point. Genes were clustered by Euclidean distance and average linkage. Individual values and probes are shown in Supplemental table 1. Also, differentially expressed lineage marker genes (from means of replicates) were calculated as relative to control/uninfected cells in both day 3-infected cells and pSLC state.

## Results

### Properties of infected non-reprogrammed and reprogrammed Schwann cells

**Figure 2.**
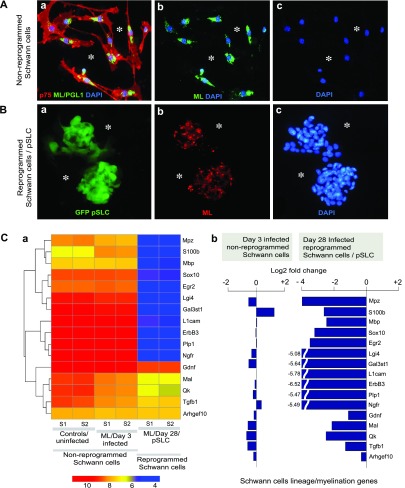
Properties of ML infected non-reprogrammed and reprogrammed Schwann cells. ( **A**) Purified adult de-differentiated Schwann cells infected with ML for 3 days and labelled with antibodies to p75NTR (red) and ML-specific PGL-1 (green), counterstained with DAPI for nuclei (blue). ( **B**) GFP pSCL (green) derived from day 28 infected Schwann cells labeled with anti-PGL1 and counterstained with DAPI (blue). Asterisks denote the absence of ML outside infected cells; ML strictly retain within the cytoplasm and no evidence of bacterial leakage to the surrounding when maintain as monocultures. Magnification: ( **A**- **a**, **b**, **c** to **B**- **a**, **b**, **c**) 20x. ( **C**) ( **a**) Expression levels of known Schwann cell lineage/myelination genes, inferred by mRNA detection by Affymetrix Mouse microarray, from 2 samples (S1 and S2) each from control/uninfected Schwann cells, infected Schwann cells for 3 days (non-reprogrammed cells preserving Schwann cell identity) and pSLC-derived cells from day 28-infected Schwann cells (reprogrammed cells with loss of Schwann cell identity). Genes are clustered by Euclidean distance and average linkage for clarity. ( **b**) Differential expression (log2 fold change) of known Schwann cell lineage markers, shown as relative to control for both day 3 infected cells (left) and 28-day-derived pSLC-state cells (right). The mean of both replicates for each of the three time points was used when calculating log2 fold change relative to control. Note that both absolute ( **a**) and differentially expressed ( **b**) patterns show a high degree of similarity between control/uninfected and day 3-infected cells, as compared to marked downregulation of Schwann lineage/in pSLC. Robust Multichip Average (RMA) values representing, on a logarithmic scale (base 2), the relative abundance of an mRNA transcript for a given gene, shown as a colour scale from highest (around 10) to lowest (around 4). Colour scale for heatmap ranges between minimum and maximum detection of selected genes, while full array's range was 2.4 to 14.2.

Schwann cells purified from mouse adult peripheral nerves maintained Schwann lineage and myelin markers and are highly susceptible to ML infection ^7^. In this study, we used Schwann cells which were infected with ML for 3 days, and previously described pSLC ^7^ which were derived from reprogrammed Schwann cells after day 28 infection. Both day 3-infected Schwann cells and pSLC showed high level of infection and strict confinement of ML to the cytoplasm ( Figure 2A and B). We found no evidence for bacterial leakage into the surrounding media when either cell type is cultured on its own. Supernatants collected from these cells after infection showed no evidence of ML in the media (data not shown). We next determine if ML infection at day 3 changed Schwann cell lineage marker expression when compared with pSLC, which are known to be reprogrammed cells exhibiting the loss of Schwann cell lineage markers ^7^. Comparative analyses revealed that infected Schwann cells at day 3 express a similar profile of Schwann cell lineage/myelination-related genes as compared to uninfected controls ( Figure 2C-a). In contrast, pSLC showed a striking downregulation of the same markers. Absolute expression profiles of Schwann cell lineage/myelination-related genes are shown in Figure 2C-a. Differential expression of day 3-infected and pSLC as compared to uninfected control cells further revealed that there is almost no change in Schwann cell identity in cells at day 3 post-infection as compared to the marked downregulation of the same genes in pSLC ( Figure 2C-b). Since pSLC, but not cells infected for 3 days, lost Schwann cell identity, we refer to pSLC and infected Schwann cells at day 3 as reprogrammed and non-reprogrammed Schwann cells respectively ( Figure 1C a, b).

### Non-reprogrammed Schwann cells possess high bacterial retention capacity in the presence of fibroblasts

**Figure 3.**
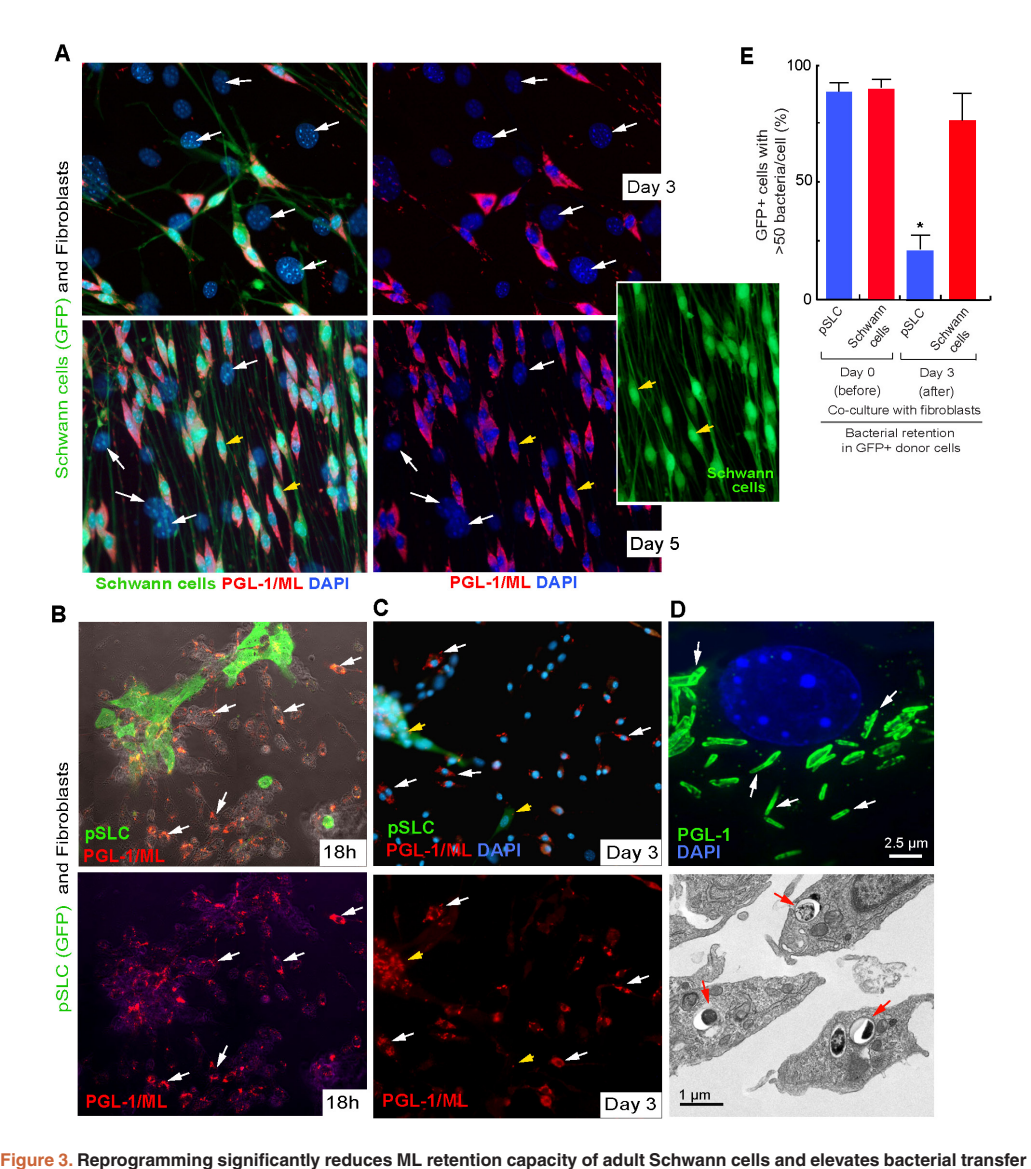
Reprogramming significantly reduces ML retention capacity of adult Schwann cells and elevates bacterial transfer property to fibroblasts ( **A**) De-differentiated Schwann cells purified from adult peripheral nerves from GFP mice were infected with ML for 3 days and incubated with fibroblasts (isolated from wild type/non-GFP peripheral nerves) for another 3 (top panel) and 5 (bottom panel) days. Fixed co-cultures were labelled with antibody to ML-specific PGL-1 (red) and nuclei were counter stained with DAPI (blue). White arrows show non-GFP fibroblasts with very few ML transferred from GFP+ Schwann cells. Note that almost all GFP+ Schwann cells carry high number of ML (yellow arrows). Inset shows GFP+ Schwann cells with typical bipolar morphology with content of ML (same as in bottom panel). ( **B**, **C**) GFP+ reprogrammed Schwann cells, pSLC effectively transfer ML to exogenously added fibroblasts. GFP-pSLC were co-cultured with fibroblasts and fixed after 18h ( **B**) and 3 days ( **C**) and labelled with anti-PGL1 antibody. In **B** (top) shows the phase-contrast image and in **C** (top) cells are counterstained with DAPI (blue). Note that numerous ML were transferred to fibroblasts within 18h; arrows in **B**, **C** show PGL-1+ ML in non-GFP fibroblasts. Yellow arrows mark the GFP+pSLC ( **B**, **C**). Magnification: ( **A**, **B**, **C**) 20x. ( **D**) Representative high-resolution confocal image (top) showing PGL-1 antibody-reactive intact rod-shaped ML (green; arrows) within fibroblasts (nucleus is labelled by DAPI; blue), and electron micrograph illustrating whole ML with electron transparent outer lipids (red arrows) in the cytoplasm of fibroblasts (bottom). ( **E**) Quantitative analysis of bacterial retention in GFP+ non-reprogrammed Schwann cells as compared to GFP+ reprogrammed Schwann cells/pSLC in the presence of fibroblasts. * < p 0.01.

The difference between non-reprogrammed and reprogrammed Schwann cells at the mRNA level correlated with their capacity to maintain or transfer ML when primary fibroblasts were introduced to these cell types ( Figure 1). GFP+ Schwann cells purified from GFP mice show high susceptibility to ML infection. When GFP+ Schwann cells at day 3 post-infection were co-cultured with non-GFP fibroblasts we found intracellular ML to be retained within the cytoplasm of Schwann cells after 3 and 5 days ( Figure 3A). Bacterial transfer to GFP negative fibroblasts was minimal even after 5 days of co-culture. Identical results were obtained regardless of the tissue type (neural or skin) from which fibroblasts were isolated and the media used for co-culturing the cells. These data suggest that non-reprogrammed Schwann cells, which preserve Schwann cell identity, expressing the full spectrum of Schwann cell lineage functional markers ( Figure 2C), retain ML in large numbers.

### Reprogramming converts Schwann cells to a stem-like cell type with effective bacterial transfer capacity

When infected GFP+pSLC were co-cultured with primary mouse fibroblasts we found that most of the ML present within pSLC were transferred to non-GFP fibroblasts within 24 hours. Figure 3B shows the transfer of PGL-1+ ML to non-GFP fibroblasts when they co-cultured for 18 hours or 3 days. Figure 1 illustrates the experimental scheme and summary of the results described below. Regardless of the origin of fibroblasts, whether they are neural fibroblasts (isolated from peripheral nerves) or dermal fibroblasts (isolated from adult skin), and regardless of choice of media for co-culture, mesenchymal media, DMEM with 6% serum or Schwann cell media, bacterial transfer assays from pSLC showed similar results. Unlike macrophages, fibroblasts are not professional phagocytic cells equipped with highly potent immune mediators capable of killing bacteria and their host cells, and thus bacterial residence in fibroblasts may provide an immune-evasion strategy for ML with a decayed genome ^13^. As expected, we could not detect any apoptotic GFP+pSLC debris phagocytosed by these fibroblasts in co-cultures. Therefore, ML transfer to fibroblasts from pSLC is unlikely to occur by an apoptosis-mediated mechanism, but mainly by a cell-to-cell transfer mechanism.

## Discussion

Results presented in this study show an example of how a bacterial pathogen could use cell fate change of its preferred host cellular niche to its own advantage during different stages of the infectious process, from bacterial colonization to bacterial transfer to fibroblasts that could facilitate the complex process of dissemination of infection. We have frequently observed that primary Schwann cells as mono-cultures retained intracellular ML for a long period within Schwann cells, regardless of their species origin, rat, human or mouse ^1, 14, 15^. Intriguingly, intracellular ML maintained Schwann cells without causing any apoptosis; this anti-apoptotic property is a defining feature of ML as compared to other pathogenic bacteria ^15, 16^. Co-culture of infected GFP+ Schwann cells with high bacterial load with fibroblasts failed to produce a significant bacterial transmission even after 5 days ( Figure 3). Such bacterial retention capacity in adult Schwann cells may also be of functional significance during human infection, since Schwann cells in leprosy patients are known to harbour ML for an extensive period, which may be critical for bacterial expansion within this privileged niche ^3, 17^. For this purpose, initial bacterial retention within Schwann cells is critical so that ML replication and colonization can be ensured. On the other hand, following sufficient intracellular propagation of ML within Schwann cells, the next step of the infectious process, as in many bacterial infections, is to transfer their progeny to a secure host cell type, which could serve as either mediator cells or vehicle that can spread the infection locally or systemically ^18^. Tissue fibroblasts could serve as non-immune mediator cell types for spreading the infection, as they are ubiquitously present in many tissues whereas macrophages, which come to action following inflammatory responses, are known to serve as a vehicle for bacterial dissemination both locally and systemically ^19^.

Once colonized, reprogramming of infected Schwann cells may be necessary for the conversion of bacterial retention capacity of parent Schwann cells to a bacterial transfer property of reprogrammed Schwann cells for effective dissemination. We have recently shown that pSLC, but not Schwann cells, effectively transfer ML to macrophages *in vivo* under inflammatory conditions ^7^. In this study, we showed that non-immune tissue cells like fibroblasts, which are much safer for ML survival than macrophages and are widely distributed (in the absence of inflammation) in peripheral nerves and skin, two preferred tissue niches for ML ^20^, are a likely target for mediating bacterial dissemination. Effective ML transfer to neural fibroblasts is of particular significance, since neural fibroblasts, which are present in the peripheral nerve microenvironment could serve as an immediate target for ML once they colonized Schwann cells and subsequently undergo reprogramming. Thus, the reprogramming of Schwann cells provide ML with ample advantages – first to colonize intact Schwann cells and then to gradually change the fate of Schwann cells to the pSLC stage, promoting transfer of bacteria to fibroblasts or perhaps to other surrounding tissue cell types. Such a strategy suggests the intriguing possibility of effective bacterial spread to a wide range of tissues via pSLC as the reprogrammed form of infected Schwann cells also acquired other essential features such as re-differentiation, and migratory and immunomodulatory properties that are highly advantageous for bacterial dissemination. Therefore, we propose that the effective ML transfer capacity of the reprogrammed form of Schwann cells to fibroblasts could be a functionally-important event during ML dissemination.

Ubiquitous distribution of fibroblasts in almost all body tissues types suggests that pathogens are most likely to take advantage of these cells in order to reach or exit from their specific tissue niches. Neural fibroblasts are abundant in peripheral nerve tissues and ML may also use these cells during the exit from Schwann cells after colonization or simply use as a safe reservoir for bacterial survival during human infection ^21, 22^. Present studies showed that primary neural fibroblasts derived from peripheral nerves could indeed serve as a susceptible recipient cell type for ML when these fibroblasts contact with infected reprogrammed Schwann cells (pSLC). The new data from our study can be extended to the conditions associated with neuropathogenesis in leprosy patients, as neural fibroblasts in leprosy patients are known to secrete and deposit extracellular matrix components, particularly collagen and causes fibrotic conditions, contributing to the irreversible nerve damage observed in leprosy ^21– 23^. Under such conditions, fibroblasts harbouring ML could serve as an additional niche for bacterial survival, persistence and spread. Indeed, in leprosy patients and nine-banded armadillos infected with ML, neural fibroblasts carrying high number of ML has been clearly demonstrated mostly in perineurial compartment ^22– 24^. Such bacterial persistence within neural fibroblasts may further perpetuate the nerve injury process by increased fibrosis and inflammation. Further molecular studies on this new role of fibroblasts in ML infection will provide novel insights into neuropathgenesis of ML infection and perhaps developing new strategies for preventing fibroblast-mediated bacterial spread from neural compartment to non-neural tissues.

The underlying mechanisms of effective bacterial retention in non-reprogrammed Schwann cells and rapid bacterial transfer capacity of pSLC are currently unknown. Based on the transcriptomic evidence from gene expression data it is possible that preserved Schwann cell identity in non-reprogammed cells and loss of Schwann cell identity in reprogrammed cells are associated with these two distinct functional properties. It is also intriguing that both non-reprogrammed and reprogrammed Schwann cells retain bacteria in the cytoplasm in the absence of exogenously added cells ( Figure 1A and B). However, rapid transfer of bacteria to exogenously added fibroblasts occurs only when pSLC interact with fibroblasts or macrophages ^7^, suggesting that signals received from recipient cells or interacting cells following cell-to-cell interaction could trigger the signals necessary for bacterial transfer process. Since apoptotic events are minimal or not detected in these pSLC and fibroblast co-culture conditions, bacterial transfer from pSLC to fibroblasts is likely mediated by non-apoptotic and cell-to-cell transfer mechanisms. Although mechanisms involving such cell-to-cell bacterial transfer process appear to be highly complex, identification of details allow for the development of strategies to ablate bacterial spread at the early stage of infection.

## Figures and Tables

**Table S1.** Microarray data for Figure 1C. Robust Multichip Average (RMA) values for selected Schwann cell lineage markers, representing absolute gene expression as calculated across 6 microarrays, from 2 samples (S1 and S2) each from control/uninfected Schwann cells, infected Schwann cells for 3 days (non-reprogrammed cells preserving Schwann cell identity) and pSLC-derived cells from day 28-infected Schwann cells ^7^.

Gene and Probe ID	"Control (1)"	"Control (2)"	"Day 3 (1)"	"Day 3 (2)"	"pSLC (1)"	"pSLC (2)"
"Tgfb1 (1420653_at)"	8.875085087	8.750895157	8.240683025	8.151701667	7.441773105	7.59125912
"Erbb3 (1434606_at)"	10.37548022	10.32681125	10.3069489	10.18805898	3.557050331	4.095306358
"Sox10 (1424985_a_at)"	8.771069925	8.756508626	8.740748716	8.70056877	5.645300737	5.467906393
"Plp1 (1425468_at)"	10.49937908	10.52663114	10.21998482	10.33987413	5.077788336	4.995512713
"Ngfr (1454903_at)"	10.42934191	10.26776964	10.75528146	10.66659223	4.974116673	4.74094638
"Lgi4 (1434121_at)"	9.391434237	9.35234632	9.014339856	9.056992656	4.786829187	3.791313289
"Mal (1432558_a_at)"	8.920254485	8.986191443	8.414628435	8.297979346	6.728140535	6.916327294
"Mpz (1423253_at)"	8.256161295	8.381011338	7.845112469	7.694736612	4.295568611	4.391234401
"S100b (1434342_at)"	6.899266755	7.029193803	8.242286924	8.204423493	4.173923744	4.505750456
"Qk (1425597_a_at)"	9.012841737	9.022546403	8.359367418	8.287088133	6.721881849	6.317931293
"Arhgef10 (1452302_at)"	7.921453062	7.896197547	7.64605098	7.766296023	7.576333594	7.610272438
"L1cam (1450435_at)"	11.22766628	11.33330355	11.21190865	11.35254211	5.658247758	5.340342385
"Egr2 (1427683_at)"	8.522475217	8.506068104	8.57181597	8.524460792	4.891965037	5.151886088
"Mbp (1419646_a_at)"	7.517747967	7.436965949	7.489265636	7.554494302	4.718879815	5.300037843
"Gal3st1 (1454078_a_at)"	10.04534115	9.950345605	9.470080807	9.457810701	4.324986565	4.37275971
"Gdnf (1419080_at)"	9.972677432	9.918438134	9.802476063	9.741177444	8.832869717	8.826992706
