# Supplementary material for: Reprogramming diminishes retention of Mycobacterium leprae in Schwann cells and elevates bacterial transfer property to fibroblasts
Source: F1000Res. 2013 Nov 14;2:198. Originally published 2013 Sep 25. [Version 3] doi: 10.12688/f1000research.2-198.v3 (PMC3829123; doi:10.12688/f1000research.2-198.v3)
Supplement: Peer review history

# Peer review: Referee response for version 3

**Published:** 2013-11-20

**Authors:** Tom Gillis

**Affiliations:** 1 Departments of Microbiology, Immunology and Parasitology, Louisiana State University School of Medicine, New Orleans, LA, USA

The authors have improved the manuscript by addressing my concerns regarding their model of neuropathogenesis and how it relates to current clinical and histopathological findings in leprosy.

I have read this submission. I believe that I have an appropriate level of expertise to confirm that it is of an acceptable scientific standard.

# Peer review: Referee response for version 3

**Published:** 2013-11-19

**Authors:** Yoshiko Takahashi

**Affiliations:** 1 Department of Zoology, Kyoto University, Kyoto, Japan

I have read this submission. I believe that I have an appropriate level of expertise to confirm that it is of an acceptable scientific standard.

# Peer review: Referee response for version 2

**Published:** 2013-11-06

**Authors:** Tom Gillis

**Affiliations:** 1 Departments of Microbiology, Immunology and Parasitology, Louisiana State University School of Medicine, New Orleans, LA, USA

The studies described herein by Masaki *et al*., extend earlier *in vitro* modeling of *M. leprae *- Schwann cell interactions by this group that may be at the crux of neurological changes and various pathogenic elements in leprosy. Specifically, this work describes phenotypic and transcriptome changes associated with SC reprogramming following long-term *M. leprae *(ML) infection that are associated with retention, or loss of retention, of ML in coculture with fibroblasts. The loss of retentive behavior is measured by the accrual of ML in fibroblasts placed in coculture with ML-infected normal or reprogrammed SC’s.

 

These studies attempt to recapitulate events occurring *in vivo* and must always be appreciated within the view of what is observed in the disease both at the clinical and histopathological level. To that end it would be helpful for the authors to:

1. Place the phenomenon of “fibroblast infection” within the context of what is seen in clinical disease with a few pertinent references.
   
2. Broaden the Discussion section to include how the author’s hypothesis of early dissemination of ML from infected pSLC (intraneural compartment) fits with histopathological observations in lepromatous leprosy infections (man and armadillo) which demonstrate a much higher bacterial burden in the extraneural compartment as compared to the intraneural compartment.

 

Finally, since fibroblasts can take up *M. leprae* in culture and infected fibroblasts have been demonstrated in tissues of infected individuals and animals, I’m wondering whether the investigators did studies with ML and fibroblasts alone. Depending on the outcome of these studies, some light might be shed on whether the transfer to fibroblasts is actually an active process (e.g., cell-to-cell contact required) or passive in nature. If data of this nature is available, it would enhance the investigator’s assertion that the reprogramming of SC’s and loss of bacterial retention is an acquired property that leads to efficient bacterial transfer to fibroblasts. Please expand on your position in the discussion to clarify.

I have read this submission. I believe that I have an appropriate level of expertise to confirm that it is of an acceptable scientific standard, however I have significant reservations, as outlined above.

# Peer review: Referee response for version 2

**Published:** 2013-11-06

**Authors:** Yoshiko Takahashi

**Affiliations:** 1 Department of Zoology, Kyoto University, Kyoto, Japan

This study is a follow up of their previous discovery published in Cell, 2013. The authors have described differences between non-programmed and programmed Schwann cells in their ML-retaining properties. Unveiling these differences is an important step toward understanding how ML disseminates in the body. The experiments were nicely done and the conclusion the authors tried to draw is justified.

I have one comment that must not be a big burden for the authors. This study deals with several different types of cells, donor primary fibroblasts, non-programmed Schwann cells, programmed Schwann cells, recipient fibroblasts derived from multiple origins etc. In addition, Schwann cells (either non-programmed or programmed) are considered either “recipient” or “donor” dependent on which way ML bacteria translocate. So, the story is a bit confusing for readers. My strong suggestion is that the authors might be willing to put one more figure in which the experimental scheme is summarized in a simple diagram. In this way, fewer readers would need to repeatedly read the same sentences before getting points of what authors want to describe.

The authors have properly addressed the comments in the previous report, which has improved the paper.

I have read this submission. I believe that I have an appropriate level of expertise to confirm that it is of an acceptable scientific standard.

# Peer review: Referee response for version 2

**Published:** 2013-11-05

**Authors:** Maximiliano Gutierrez

**Affiliations:** 1 MRC National Institute for Medical Research, London, UK

I thank the authors for addressing and clarifying my specific comments. I appreciate the details that are now provided in the new version and I do not have further comments.

I have read this submission. I believe that I have an appropriate level of expertise to confirm that it is of an acceptable scientific standard.

# Peer review: Referee response for version 1

**Published:** 2013-10-10

**Authors:** Maximiliano Gutierrez

**Affiliations:** 1 MRC National Institute for Medical Research, London, UK

This paper reports an intriguing aspect of the complex pathogenesis of the obligatory intracellular bacteria *M. leprae (ML)*. The authors report that ML infecting Schwann cells are not able to disseminate to fibroblasts but the cells re-programmed by the bacteria in stem-cell like cells are efficiently transferred to the same cells *in vitro*. These observations are very interesting and contribute to our understanding of the phenomenon of mycobacterial cell-to-cell transfer.

 

There are only three technical points I think that authors could consider in this work, to unambiguously claim that the putative cell-to-cell transfer mechanism is a non-apoptotic, non-lytic one:

1. The authors stated as data not shown that there is not evidence of bacteria in supernatants. It would be important to show this information and indicate in the methods how it was performed.
2. The authors mentioned that they could not detect any apoptotic GFP+pSLC debris. It would be necessary to show these experiments as well, to exclude e.g. efferocytosis. They should indicate in the methods how apoptosis was detected (markers, staining etc…).
3. The bacterial transfer to fibroblasts is measured primarily by detection of one lipid, PGL-1. It is known that lipids from *M. tuberculosis* can be transferred from infected cells to “bystander” cells ( Beatty WL et al. 2000). I understand that the system with ML is much more complicated, but would it be possible to confirm that the fluorescent signal represent ‘intact’ bacteria e.g by electron microscopy?

 

A minor comment: the authors refer to “strict confinement of ML to the cytoplasm” (results) or “retain bacteria in the cytoplasm” (discussion). Do they mean intracellular?

I have read this submission. I believe that I have an appropriate level of expertise to confirm that it is of an acceptable scientific standard, however I have significant reservations, as outlined above.

# Response

**Published:** 2013-10-17

**Authors:** Anura Rambukkana

**Affiliations:** University of Edinburgh, UK

We thank the reviewer for the constructive and valid comments. We note here that because of the short nature of this article we did not describe all negative results and present additional data in the original version. Here we address and clarify these issues:

 

1. Evidence for the absence of bacteria in the supernatant and how it was performed is now elaborated in the methods section of the revised manuscript.
   
2.  As described in our previous reports ( Masaki *et al*., 2013), ( Tapinos and Rambukkana, 2005) apoptosis was measured using TUNNEL assay. It is known that *M. leprae* infection does not induce apoptosis ( Lahiri *et al*., 2010), and in the present study no increase in *M. leprae* TUNNEL-positive cells were detected in infected co-cultures as compared to uninfected cells.
   
3. Phenolic glycolipid-1 (PGL-1), particularly its sugar moieties, is very unique to *M. leprae* and is present in high quantity in the *M. leprae* cell wall. The latter permits the detection of intact *M. leprae* using antibodies against PGL-1. We have used a well-characterized antibody (IgG) against the native sugar/lipid moieties of PGL-1 that has been widely used for detecting whole/intact *M. leprae* in infected cells and tissues ( Ng *et al*., 2010; Masaki *et al.*, 2013). PGL-1 antibody activity has also been shown to correlate with acid-fast labeling (Fite’s staining) that specifically stains intact mycobacteria in infected tissues ( Masaki *et al*., 2013). Considering the specificity and the detection of rod-shaped bacteria in high numbers in non-GFP fibroblasts co-cultured with pSLC, it was concluded that anti-PGL1 antibody detect the intact *M. leprae*, but not free lipids. Moreover, high load of *M. leprae* in non-reprogrammed Schwann cells (Fig. 2A), reflects the potential high content of bacterial lipids in the cytoplasm, but we failed to detect any significant PGL positivity in fibroblasts co-cultured with non-reprogrammed Schwann cells (Fig. 2A). This further suggests a lack of active (or significant) bacterial lipid transfer from one cell to another under these experimental conditions.
  Nevertheless, to further clarify this point the updated version includes a representative high-resolution fluorescence image of intact rod-shaped bacteria in fibroblasts detected by anti-PGL-1 antibody. In addition, we also include a representative electron micrograph showing the presence of intact *M. leprae* in fibroblasts (Fig. 2B). It should be noted that infected fibroblasts are distinguishable from pSLC under the electron microscopy, since pSLC, but not fibroblasts, form cell aggregates/clusters (Fig. 1a).
   
4. Minor comment: here we emphasize that all *M. leprae* detection is strictly intracellular.
